# Semilobar holoprosencephaly in a 12-month-old baby boy born to a primigravida patient with type 1 diabetes mellitus: a case report

**DOI:** 10.1186/s13256-016-1141-y

**Published:** 2016-12-20

**Authors:** Pedro Pallangyo, Frederick Lyimo, Paulina Nicholaus, Hilda Makungu, Maria Mtolera, Isaac Mawenya

**Affiliations:** 1 Unit of Research, Department of Cardiovascular Medicine, The Jakaya Kikwete Cardiac Institute, PO Box 65141, Dar es Salaam, Tanzania; 2 Department of Radiology, Muhimbili National Hospital, PO Box 65000, Dar es Salaam, Tanzania; 3 Department of Pediatric Cardiology, Jakaya Kikwete Cardiac Institute, PO Box 65141, Dar es Salaam, Tanzania

**Keywords:** Holoprosencephaly, Semilobar holoprosencephaly, Structural brain malformation, Developmental delay, Case report

## Abstract

**Background:**

Holoprosencephaly is a rare spectrum of cephalic disorders resulting from a failure or incomplete division of the embryonic forebrain into distinct cerebral hemispheres. It is the most common brain malformation with an incidence of 1:250 during embryogenesis; however, owing to the associated high rates of spontaneous abortion the incidence is 1:16,000 among live deliveries. Pathogenesis of holoprosencephaly is complex and heterogeneous involving genetic abnormalities, teratogenic exposures, and syndromic associations. Among the teratogenic exposures, maternal diabetes is a well-established risk factor associated with a 200-fold increased incidence of holoprosencephaly.

**Case presentation:**

We report a case of a delayed diagnosis of semilobar holoprosencephaly in a 12-month-old baby boy of African descent who presented to us with a history of global developmental delay, erratic sleep patterns, and poor weight gain. He was born to a type 1 diabetes mellitus mother at 39+ weeks by emergency cesarean section due to fetal distress and breech presentation. The baby weighed 2315 g and had Apgar scores of 6/10 and 8/10 at 1 and 5 minutes respectively. A physical examination done at 12 months of age revealed a small-for-age child with a developmental age of 2 months. He had normal facies but a neurological examination revealed hypotonia in all four limbs. The rest of systemic examination was unremarkable. Hematological and biochemical investigations revealed normal findings except for iron deficiency anemia. The child also underwent magnetic resonance imaging of his brain which revealed distinctive features of semilobar holoprosencephaly. He was treated for iron deficiency anemia with Hemovit syrup (ferric ammonium citrate, folic acid, pyridoxine hydrochloride, cyanocobalamin, and zinc sulfate) 10 ml thrice daily, ferrous sulfate 10 mg once daily, folic acid 1 mg once daily, and multivitamin syrup 5 ml once daily. Furthermore, nutritional and genetic counseling was offered to his parents.

**Conclusions:**

In conclusion, although rare, holoprosencephaly is the commonest structural anomaly of the brain with a complex and multifactorial etiopathogenesis. It is prudent to diagnose it prenatally, classify its severity, and forge its prognosis so that parents are counseled early enough to make informed decisions especially where termination of pregnancy may be implicated.

## Background

Congenital structural malformations of the central nervous system are responsible for a considerable morbidity and mortality in childhood with resultant long-term psychological, social, and financial implications for the survivors and their families. Holoprosencephaly (HPE) encompasses a continuum of brain malformations characterized by a failed or incomplete cleavage of the embryonic forebrain into distinct cerebral hemispheres often occurring by the fourth week of gestation [1–8]. It is the most common brain malformation with an incidence of 1:250 during embryogenesis; however, owing to the associated high rates of spontaneous abortion the incidence of HPE is 1:16,000 among live deliveries [3–8]. Classically, HPE is categorized into three main subtypes in order of decreasing severity: alobar, semilobar, and lobar.

The etiopathogenesis of HPE is multifactorial involving the interplay of various environmental and genetic factors. Neurodevelopmental delay is present in virtually all affected children while other manifestations including hypotonia, seizures, aspiration pneumonia, pituitary dysfunction, and failure to thrive are not uncommon [6, 8]. Owing to a shared embryonic origin between the forebrain and midface (that is, prechordal mesoderm), over three-quarters of individuals with HPE have concomitant craniofacial anomalies some of which are incompatible with life (for example, cyclopia) [2, 6, 8]. Diagnosis of HPE is usually made by prenatal transabdominal ultrasound or a computed tomography (CT) scan/magnetic resonance imaging (MRI) and its management is basically supportive. Survival and developmental outcome is generally poor and is dictated by the HPE subtype and the degree of the accompanying craniofacial dysmorphism [8]. We report a case of semilobar HPE in a 12-month-old baby boy of African descent who was born to a mother with type 1 diabetes mellitus.

## Case presentation

A 12-month-old baby boy of African descent presented to us with a history of global developmental delay, erratic sleep patterns, and poor weight gain. He was the first born to a 28-year-old woman with type 1 diabetes mellitus diagnosed at the age of 9. The mother attended antenatal clinic from the 16th week of gestation and received all routine antenatal care as per the Tanzanian protocol (antihelminthics, antimalarial medications, hematinics, and tetanus toxoid). She tested negative for human immunodeficiency virus (HIV), hepatitis B and C, and syphilis. The mother denied history of toxoplasmosis, other (syphilis, varicella-zoster, parvovirus B19), rubella, cytomegalovirus, and herpes (TORCH) infections, trauma, or chronic drug use; however, she had a urinary tract infection during her first trimester which was successfully treated with oral cephalexin 500 mg four times a day for 5 days. Apart from the insulin-dependent diabetes mellitus, no other history of familial genetic disorders was elicited. Sonographic evaluation at 16th and 36th week of gestation revealed a normal singleton pregnancy. Despite a fairly good sugar control before pregnancy, the mother had poor glycemic control during pregnancy and her glycated hemoglobin (HbA_1C_) taken at 7 and 29 weeks of gestation was 8.3% and 7.9% respectively. She gave birth at 39+ weeks by emergency cesarean section due to fetal distress and breech presentation. The baby weighed 2315 g and had Apgar scores of 6/10 and 8/10 at 1 and 5 minutes respectively. His head circumference at birth was 38.9 cm (97th percentile). Chromosome analysis (karyotyping) is not a routine practice in Tanzania and it was not performed.

**Fig. 1:**
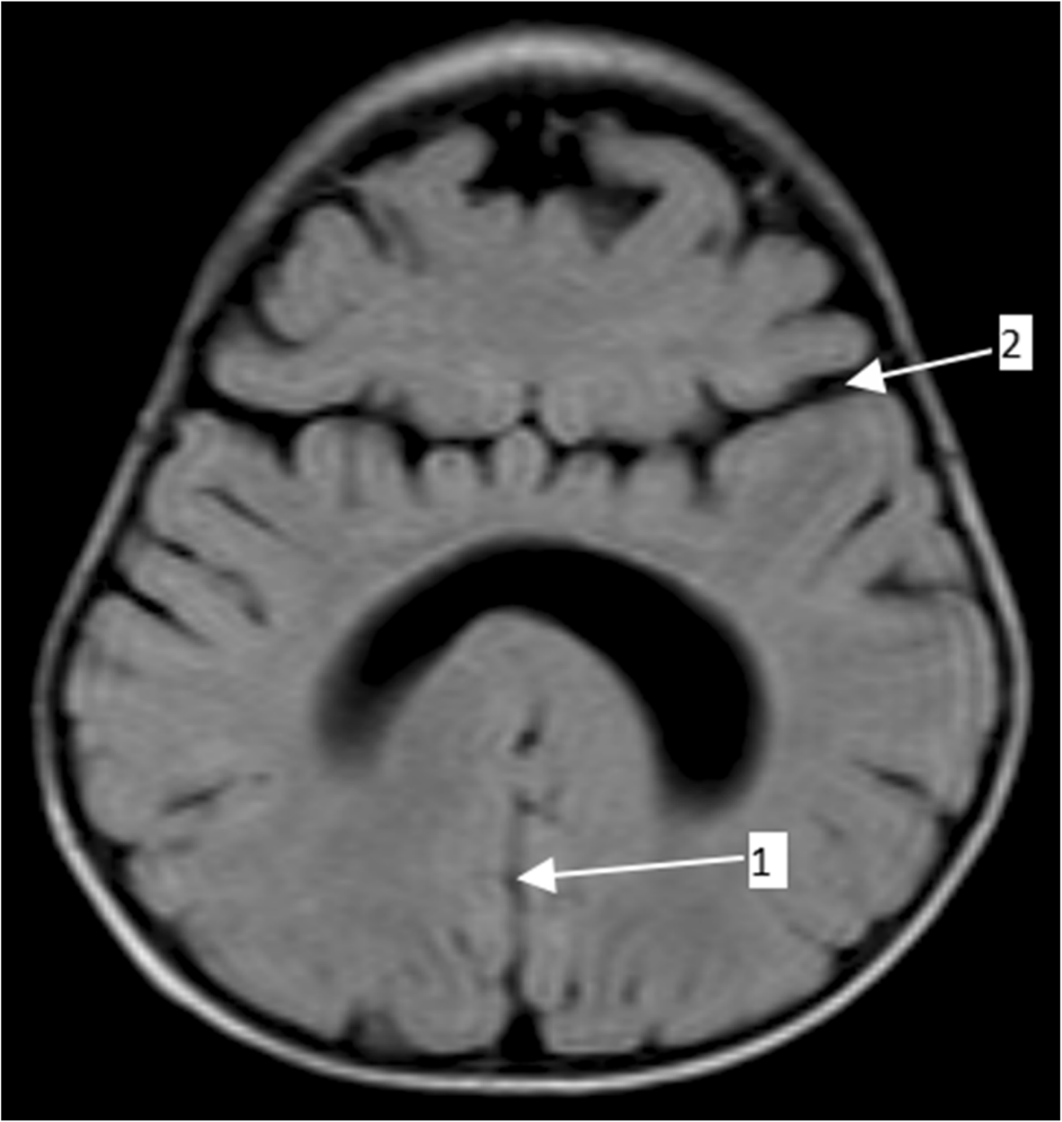
Brain magnetic resonance imaging (fluid-attenuated inversion recovery) displaying an incompletely formed interhemispheric fissure (*arrow 1*) and partial fusion of the frontal lobe (*arrow 2*)

**Fig. 2:**
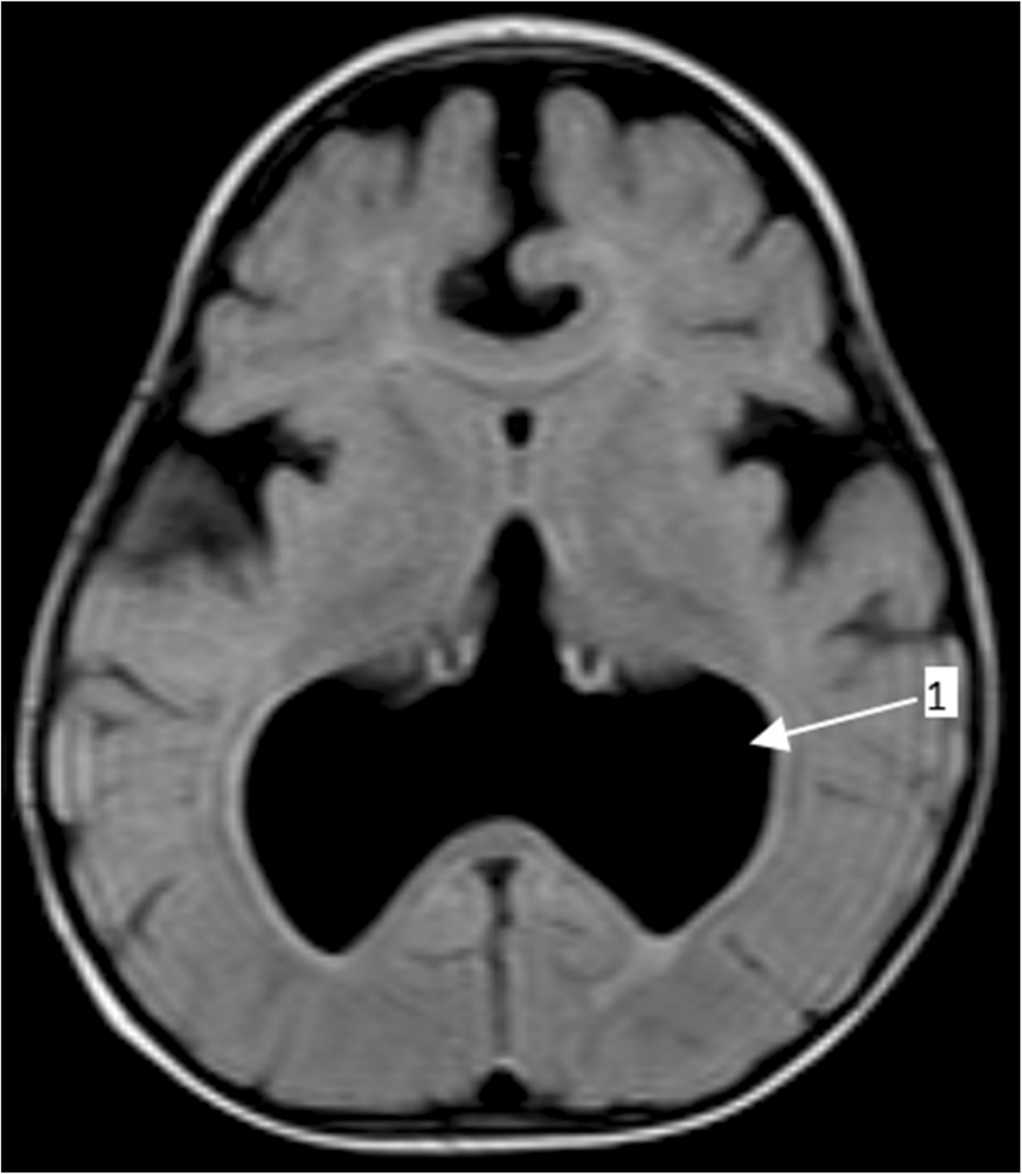
Brain magnetic resonance imaging (fluid-attenuated inversion recovery) displaying a mono ventricle with partially developed temporal and occipital horns (*arrow 1*)

**Fig. 3:**
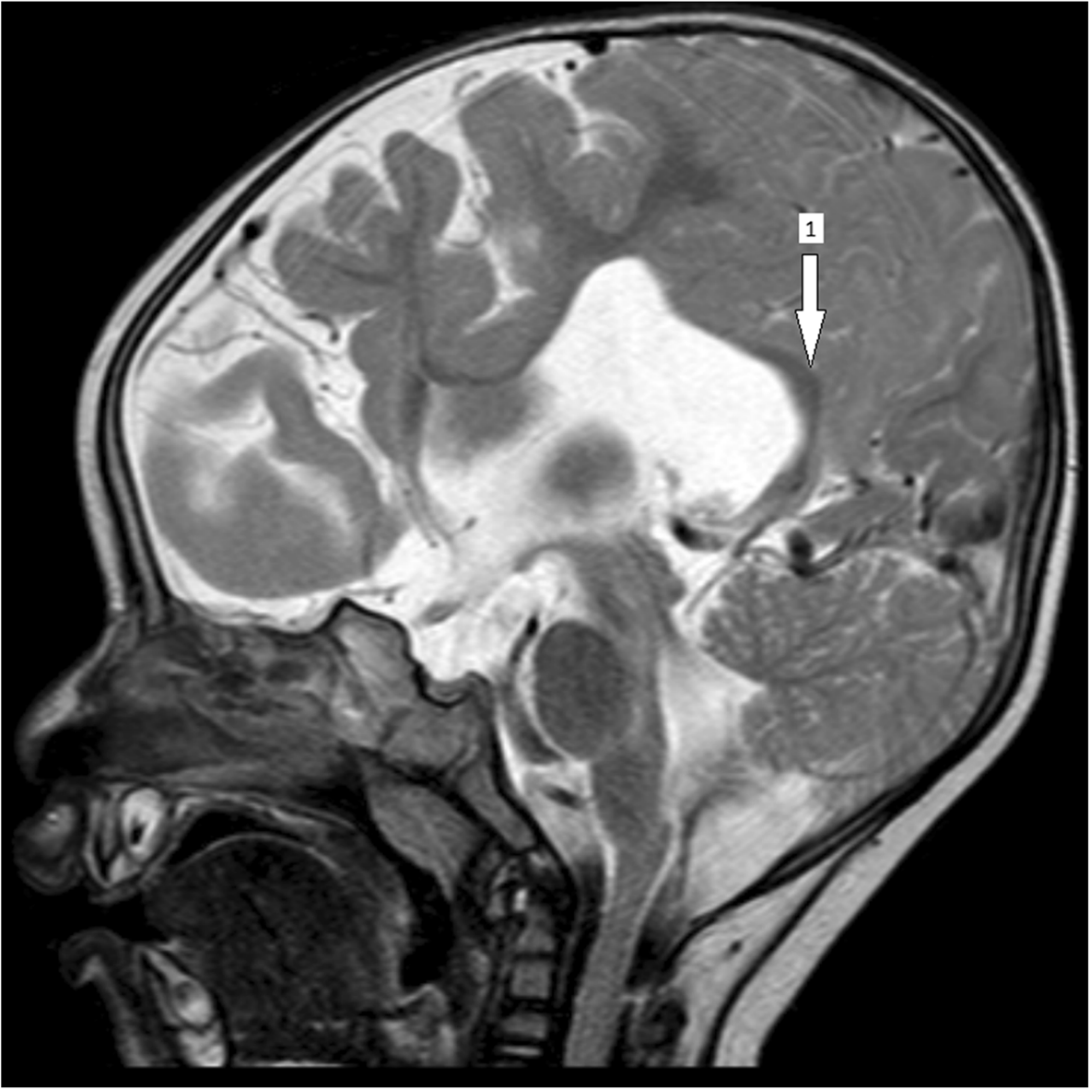
Brain magnetic resonance imaging (T2 weighted) displaying a partially formed corpus callosum (*arrow 1*)

The boy weighed 8.5 kg at 6 months, which was the exact same weight measured during his visit to our institution at 12 months of age. His head circumference at 1 year was 49.1 cm (99th percentile). He had stable vital signs but a physical examination revealed a small-for-age child with conjunctival and palmar pallor. All his growth parameters were below the 5th percentile and he had a developmental age of 2 months. He scored 4 on neurocognitive assessment using the Carter Neurocognitive Assessment Severity Scale. He had normal facies but a neurological examination revealed hypotonia in all four limbs. Other systemic examinations were unremarkable. He underwent a series of blood work-up, cardiac echocardiography (ECHO), and renal ultrasound all of which revealed normal findings except for iron deficiency anemia: hemoglobin (Hb) 8.7 g/dL, mean corpuscular hemoglobin (MCH) 21 pg/cell, mean corpuscular volume (MCV) 69 fL, and red cell distribution width (RDW) 15.1%. The child also underwent MRI of his brain which revealed an incompletely formed interhemispheric fissure, a monoventricle with partially developed temporal and occipital horns, partial fusion of his frontal lobe, hypoplastic corpus callosum, and volume loss bilaterally at temporal lobes (Figs. 1, 2, and 3). His cerebellum and brain stem appeared normal. Based on the clinical presentation and MRI findings the diagnosis of semilobar HPE was entertained. The child was treated for iron deficiency anemia with Hemovit syrup (ferric ammonium citrate, folic acid, pyridoxine hydrochloride, cyanocobalamin, and zinc sulfate) 10 ml thrice daily, ferrous sulfate 10 mg once daily, folic acid 1 mg once daily, and multivitamin syrup 5 ml once daily. Furthermore, nutritional and genetic counseling was offered to his parents.

The child continued to be attended by a developmental pediatrician and pediatric dietician on a regular basis at our institution. An assessment conducted at 12 months post-initial visit showed improvement in his neurocognitive status (Carter Neurocognitive Assessment Severity Scale score = 2). Furthermore, he had attained an acceptable weight for his age (12.6 kg) and his sleeping pattern was stable. In the long run, he is scheduled for both physical and occupational therapy aiming to enhance his motor skills and maximize functional independence.

## Discussion

HPE is a rare spectrum of cephalic disorders resulting from a failure or incomplete division of the embryonic forebrain into distinct cerebral hemispheres. In the semilobar HPE subtype, the cerebral hemispheres separate posteriorly but not anteriorly. The pathogenesis of HPE is complex and heterogeneous involving genetic abnormalities [9–11], teratogenic exposures [12–17], and syndromic associations [18–23]. Among the teratogenic exposures, maternal hyperglycemia is an established risk factor for HPE [1, 8, 24]. Barr *et al*. found a 200-fold increased risk for HPE among babies born to diabetic mothers compared to those of diabetes-free mothers [24]. In the case presented, the mother was known to have insulin-dependent diabetes mellitus and her glycemic control during pregnancy was poor. Long-term use of drugs including aspirin [12], statins [13], misoprostol [14], methotrexate [15], retinoic acid [16], and alcohol [17] have shown increased risk in the animal model, results which are yet to be replicated in humans [1, 8]. However, in the case presented the mother denied chronic use of medications apart from insulin injections for her diabetes. Moreover, TORCH infections early in pregnancy may potentially affect cerebral embryogenesis leading to HPE [1, 8]; a possibility of such infections in our case was, however, ruled out. Although craniofacial anomalies frequently coexist with HPE, they are usually mild or absent in the semilobar subtype as was witnessed in this case. Such anomalies include microcephaly, single central maxillary incisor, cleft lip and palate, flat nose, absent nasal bridge, microphthalmia, absence of lateral philtral ridges, absence of the superior lingual frenulum, iris coloboma, and cyclopia [2, 5, 7, 8].

Diagnosis of HPE is usually made prenatally in women with a high index of suspicion. Our case, however, was not diagnosed until the age of 1 year despite evidence of hyperglycemia exposure *in utero* and infancy symptomatology suggestive of HPE. However, HPE prognosis remains generally poor, with the type of HPE and coexisting craniofacial anomalies as key prognostic indicators. For instance, less than 20% of patients with the alobar subtype will survive to 12 months while approximately 50% with the isolated semilobar form will survive beyond 1 year [3]. Management of HPE although mainly supportive requires a multidisciplinary approach aiming at managing symptoms and complications, and avoiding added disability to improve the overall quality of life. This case despite its typical presentation was actually the first case of HPE to be diagnosed and documented in Tanzania. It is the authors’ hope that upon its publication this case report will sensitize practitioners especially in resource poor settings to raise an index of suspicion for HPE particularly in women of high risk.

## Conclusions

In conclusion, although rare, HPE is the commonest structural anomaly of the brain with a complex and multifactorial etiopathogenesis. It is prudent to diagnose it prenatally, classify its severity, and forge its prognosis so that parents are counseled early enough to make informed decisions especially where termination of pregnancy may be implicated. Furthermore, in cases with somewhat better prognosis, thorough counseling is crucial to prepare expectant parents to understand, accept, and prepare for the challenges in raising a child with HPE.

## Abbreviations

CT: Computed tomography
ECHO: Echocardiography
Hb: Hemoglobin
HbA_1C_: Glycated hemoglobin
HPE: Holoprosencephaly
MCH: Mean corpuscular hemoglobin
MCV: Mean corpuscular volume
MRI: Magnetic resonance imaging
RDW: Red cell distribution width
TORCH: Toxoplasmosis, other (syphilis, varicella-zoster, parvovirus B19), rubella, cytomegalovirus, and herpes
